# Cell Therapy in Idiopathic Pulmonary Fibrosis†

**DOI:** 10.3390/medsci6030064

**Published:** 2018-08-13

**Authors:** Anna Serrano-Mollar

**Affiliations:** 1 Departamento de Patología Experimental, Instituto de Investigaciones Biomédicas de Barcelona IIBB-CSIC-IDIBAPS, Rosselló, 161, 08036 Barcelona, Spain; anna.serranomollar@iibb.csic.es; Tel.: +34-933-638-307; 2 Centro de Investigaciones Biomédicas en Red de Enfermedades Respiratorias (CIBERES), Melchor Fernández Almagro 3, 28029 Madrid, Spain

**Keywords:** cell therapy, stem cells, lung progenitor cells, preclinical studies, bleomycin, pulmonary fibrosis, clinical studies, idiopathic pulmonary fibrosis

## Abstract

Idiopathic pulmonary fibrosis is a fatal disease with no effective or curative treatment options. In recent decades, cell-based therapies using stem cells or lung progenitor cells to regenerate lung tissue have experienced rapid growth in both preclinical animal models and translational clinical studies. In this review, the current knowledge of these cell therapies is summarized. Although further investigations are required, these studies indicate that cell therapies are a promising therapeutic approach for the treatment of idiopathic pulmonary fibrosis.

## 1. Introduction

Idiopathic pulmonary fibrosis (IPF) is a chronic, progressive and severe disease with no known cause. Damage to the alveolar epithelium is believed to be an important early pathogenic event in IPF. Epithelial cell damage and death result in gaps in epithelial basement membranes. The migration of fibroblasts and myofibroblasts into the alveolar space through these gaps leads to intra-alveolar fibrosis with an exaggerated accumulation of extracellular matrix (ECM) components. This fibrosis disturbs the normal lung architecture, leading to lung dysfunction and failure [1,2,3]. To date, pirfenidone and nintedanib are the only drugs approved for the treatment of IPF. Although these two drugs have shown efficacy in reducing the rate of decline in lung function and slowing the pace of disease progression, they are not able to halt disease progression [4,5]. Currently, it is not clear if pirfenidone or nintedanib can increase survival or improve symptoms such as dyspnoea and cough [4,5]. In addition, both compounds have shown significant side effects and neither is curative [4,5]. Due to the few options available for the treatment of IPF patients, there is a search for new therapeutic approaches. In this sense, over the last decade, several studies related to cell therapies have been conducted in both animal models and in patients with IPF in order to find new effective therapies for this devastating disease (Table 1).

The implantation of cells with the ability to proliferate and migrate to injured sites combined with the capacity to secrete multiple paracrine factors that can regulate endothelial and epithelial permeability, decrease inflammation, inhibit bacterial growth, and enhance tissue repair, is the main objective of these cell therapies for the treatment of IPF. To reach this goal, many different cell types have been assayed, including stem cells and lung progenitor cells. This review focuses on the main cells therapies used in preclinical and clinical studies.

## 2. Stem Cells

**Figure 1:**
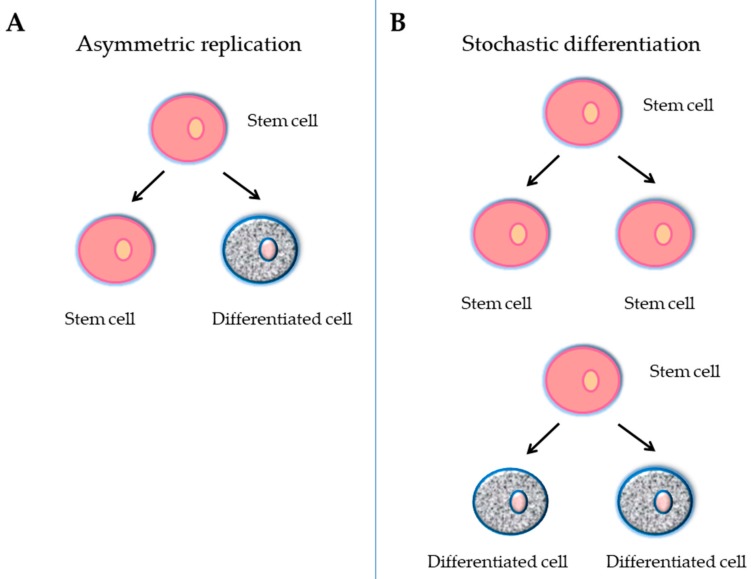
Schematic representation of a stem cell division in relation to self-renewal and the repopulation potential. (**A**) Asymmetric replication, giving rise to a differentiating cell and a stem cell; this division maintains the stem cell pool; (**B**) Stochastic model of division, giving rise to two stem cells with higher repopulation potential or to two differentiated cells.

**Figure 2:**
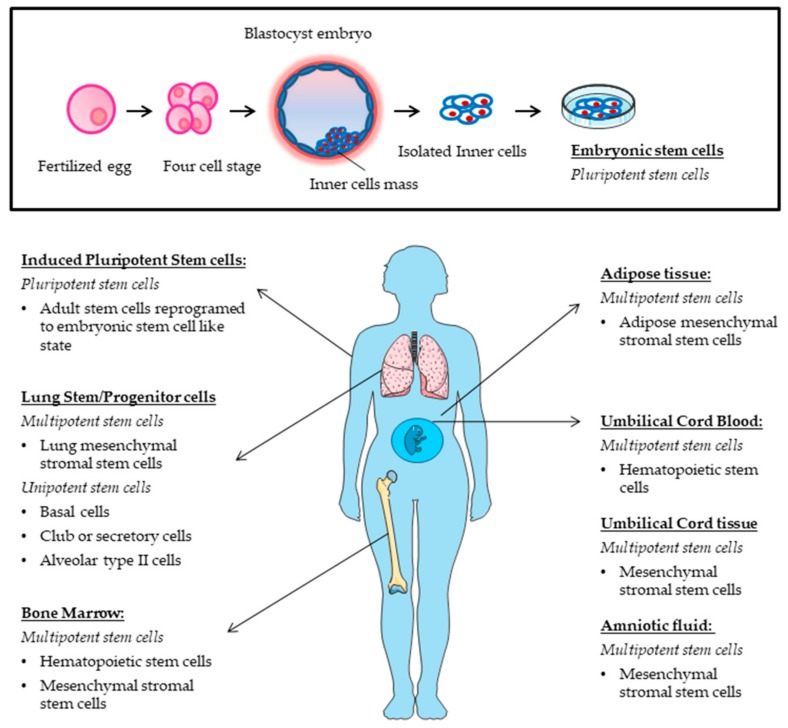
Schematic representation of the main sources for stem cells that have been used for the development of cellular therapies in pulmonary fibrosis.

A stem cell is defined as an undifferentiated cell with three primary functions: self-renewal, clonality and the potential to differentiate into different types of cells and tissue. To achieve this remarkable task, they can undergo an intrinsically asymmetric cell division whereby in the first division one daughter cell is maintained as a self-renewing stem cell and the other becomes a precursor or progenitor cell that will give rise to differentiated cells (Figure 1A). Alternatively, the stochastic differentiation process can take place. In this case, the divided stem cell could be differentiated into two daughter cells, or the stem cell could be divided into two new stem cells (Figure 1B). In accordance with the ability to differentiate, stem cells can be categorized into five groups: totipotent, pluripotent, multipotent, oligopotent, and unipotent [39]. The classification of stem cells also depends on their origin: embryonic stem cells (ESCs), adult stem cells (ASCs), and adult specific cells that have been "reprogrammed" genetically to adopt a stem cell-like state (Figure 2). This last type of stem cells is called induced pluripotent stem cells (iPCs) (Figure 2).

### 2.1. Embryonic Stem Cells

Embryonic stem cells derived from blastocysts are self-renewable and pluripotent cells that generate a variety of specialized cell types including pulmonary cells (Figure 2) [40]. Advances in lung regeneration or repair using ESCs have developed more slowly than expected, since obtaining these cells has historically involved the destruction of embryos with the obvious ethical issues. Moreover, the protocols for differentiating ESCs into lung cells have not been very accurate, although new protocols are now available to obtain differentiated lung cells from ESCs [41].

### 2.2. Adult Stem Cells

#### 2.2.1. Bone Marrow Stem Cells

In the bone marrow (BM), there are two main populations of stem cells: the hematopoietic stem cells (HSCs) and the mesenchymal stromal stem cells (MSCs) (Figure 2).

- Hematopoietic stem cells

Hematopoietic stem cells are recognized as the main source of adult stem cells with the ability to self-renew and to differentiate into all blood lineages. Hematopoietic stem cells are multipotent and they differentiate into several cell types, including endothelial [42] and epithelial cells [43]. Some groups have observed that, in the lung, HSCs are able to become alveolar type II (ATII) cells after BM transplantation [44,45,46].

- Mesenchymal stromal stem cells

Mesenchymal stromal stem cells are non-blood adult stem cells that were originally isolated from BM stroma and described by Friedenstein in 1968 [47]. Thereafter, MSCs can be isolated from other tissues, such as adipose tissue, placenta, umbilical cord, amniotic fluid and cord blood (Figure 2). Mesenchymal stromal stem cells are multipotent cells capable of differentiating into a number of different cell lines and exerting anti-proliferative, immune-modulatory, anti-inflammatory, anti-fibrotic and microbicide effects [48]. Mesenchymal stromal stem cells have the ability to home to sites of injury regardless of the route of administration [49,50]. Due to the combination of multipotency, migratory ability, and immunoprivileged state (MSCs do not express major histocompatibility factor II, making allogeneic transplant possible) [51], MSCs have shown promise as a novel therapeutic agent in multiple diseases. Mesenchymal stromal stem cells have been the most widely studied ASCs for the treatment of pulmonary diseases. A large number of preclinical studies in pulmonary fibrosis showed that MSCs migrate to the damaged pulmonary tissue, inhibit inflammation, and could differentiate into lung epithelial cells and confer a functional benefit (Table 1).

#### 2.2.2. Induced Pluripotent Stem Cells

Induced pluripotent stem cells are adult cells that have been genetically reprogrammed to an ESC cell-like state (Figure 2). In 2006, Takahashi and Yamanaka reported for the first time the successful reprogramming of somatic cells to an ESC cell-like pluripotent state [52]. The reprogramming requires the ectopic expression of four or even fewer factors (Oct 3/4, Sox2, *c-Myc*, and Klf4) responsible for maintaining pluripotency [52,53]. To date, a great variety of human and murine terminally differentiated somatic cells have been reported to be reprogrammed into iPSCs. Simultaneously, iPSCs can differentiate into any of the cell types responsible for the formation of particular tissues and organs, including ATII-like cells [54]. The derivation of iPSCs bypasses ethical concerns associated with the use of human ESCs and also provides an alternative source of cells that can be used as donor cells for transplantation therapy.

#### 2.2.3. Lung Stem/Progenitor Cells 

Lung stem and progenitor cells have attracted significant interest, as they are able to differentiate into lung epithelial cells contributing to epithelial maintenance and injury repair [55]. The lung epithelium contains several different types of epithelial cell populations, including ciliated, goblet, and basal cells in the proximal airways, and ciliated, club (or secretory), neuroendocrine, and basal cells in the distal airways. The pseudostratified airway epithelial layer continues into a flat epithelial layer composed of ATI cells and ATII cells in the alveolar regions. Lung stem and progenitor cells are found throughout the pulmonary epithelium (Figure 2).

Basal cells are morphologically characterized by their small height and by their position attached to the basement membrane of the stratified and pseudostratified airway epithelium [56]. In recent years, basal cells have been considered stem/progenitor cells since in vitro and in vivo studies have demonstrated that these cells were able to self-renew and have multipotent properties, since they could generate not only more basal cells, but also ciliated, goblet and club cells [57,58,59]. These observations have been reaffirmed using in vivo models of injury/repair which have demonstrated that the disruption of the basal cell layer triggered an uncontrolled proliferation of the underlying stroma, resulting in an accumulation of fibroblasts and immune cells that subsequently obliterate the airways [60]. Recently, it has been revealed that there are multiple heterogeneous subpopulations of basal cells. However, in human lungs these basal cell subpopulations are still not well known. There are subsets expressing distinct keratin (KRT) isoforms. Smirnova and colleagues have identified different basal cell subtypes in healthy lungs compared with an IPF human lung, characterized by various combinations of p63, KRT5, and KRT14 expression [56]. They found several combinations of KRT5^+/−^ KRT14^+/−^ p63^+/−^ basal cells in the human airways in distinct locations; however, none with the combination of KRT5^+^, KRT14^+^, and p63^+^ were found in the healthy distal lung. In contrast, this combination KRT5^+^, KRT14^+^, and p63^+^ was the most frequent in IPF distal lungs, indicating that basal cell populations in IPF patients are functionally different to basal cells in healthy lungs. Moreover, these IPF basal cell populations express differentiated epithelial cell markers, showing that in a context of injury/disease, they are likely attempting to regenerate the epithelium [56].

Club or secretory cells are the predominant epithelial cell of the bronchioles. The club cell population at large is molecularly characterized by the expression of secretoglobin family 1A member 1 (Scgb1a1) and are known to self-renew and generate ciliated cells during epithelial homeostasis and in response to lung damage [61,62,63]. Recent studies performed in mouse lungs indicate that club cells are, like basal cells, a highly heterogeneous population. In the large airway, three subsets of club cells can be discriminated based upon their maturity [57]. Other studies found different club cell division patterns; one destined to self-renewal and the classical one destined to differentiate specifically into ciliated cells [61]. In the small airways, two subsets of club cells were first identified based on their susceptibility to naphthalene-induced injury. One of them is very sensitive to naphthalene administration and a small subset is able to resist injury [64]. These naphthalene-resistant club cells, termed ‘variant club cells’, are able to replicate and repopulate the damaged airway epithelium and they are characterized by low levels of Scgb1a1 expression, in contrast to their conventional club cell counterparts. Lineage-tracing studies have shown that the population of Scgb1A1^+^ cells in the small airways self-renews and can generated ciliated cells in the absence of a basal stem cell compartment. However, it has been suggested that most ciliated cells in the large airway come from a club cell differentiation under steady state conditions, mimicking the pattern seen in the small airways [61]. It is interesting to note that, although progenitor cells are often thought of as ‘undifferentiated’ cells, club cells are functionally differentiated cells that retain progenitor cell activity [61].

In the alveoli, ATII cells that produce pro-surfactant protein C (pro-SPC) and recycle surfactant proteins are well recognized as progenitors for the alveolar type 1 cells [65]. In the adult, lineage-tracing analysis has demonstrated that ATII cells maintain the homeostatic turnover of ATI cells and also contribute extensively to the ATI population following bleomycin (BLM)-induced lung injury [65,66,67]. Moreover, lineage-tracing analysis has also shown that ATII cells not only generate ATI cells, but can also self-renew, thus qualifying these cells as stem/progenitor cells of the adult lung alveoli [65]. Treutlein and colleagues have identified a bipotent progenitor cell population in embryos that expresses both ATII and ATI cell markers [68]. At the moment, it is unclear how to compare an adult ATII cell with this embryonic one. Along these lines, an ATII cell subset, characterized by Wnt-responsive Axin2 expression, has recently been identified, and these progenitors have a particularly robust capacity to generate alveolar epithelium [69]. Interestingly, by using cell lineage tracing methods in mice, it has been shown that club cells can also give rise to ATII and ATI cells during repair processes of the alveolar epithelium damage [70]. It is likely that further subsets of ATII cells will emerge with further scrutiny.

Currently, there is a multitude studies that have confirmed the presence of MSCs in the lungs of mice [64,65,66,67,68,69,70,71,72,73,74,75] and humans [76,77,78]. Lung MSCs (lu-MSCs) share some features with BM-MSC and fulfil the International Society of Cellular Therapy criteria for MSCs, including marker profile and differentiation capacity [79]. Lung MSCs are localized at the distal tip of the branching airway epithelium and it has been observed that they can proliferate and differentiate into airway epithelial stem/progenitor cells in co-culture [80].

## 3. Preclinical Experiences

**Figure 3:**
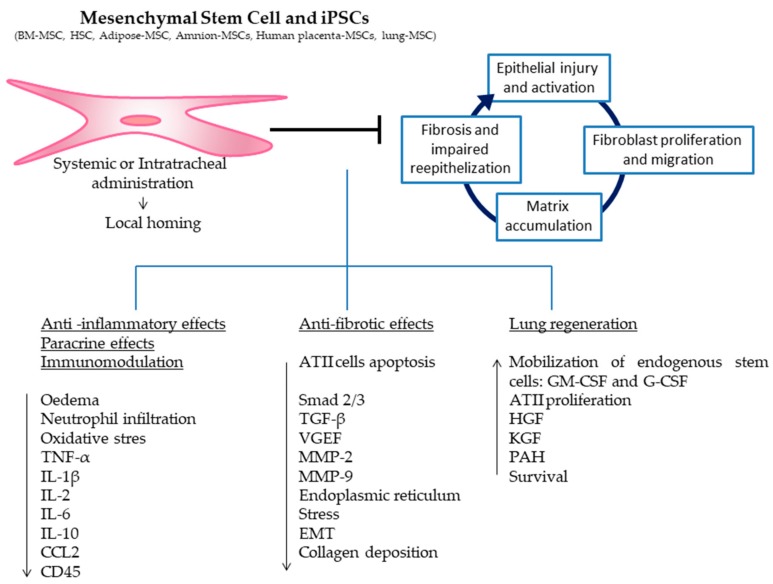
Mesenchymal stem cells or induced pluripotent stem cells delivered intravenously or intratracheally home to the sites of injury in the lungs where they exert anti-inflammatory and anti-fibrotic effects, engage in paracrine signaling and immunomodulation, differentiate into local cell types, and activate resident stem cells enhancing lung regeneration.

Numerous preclinical studies show that cell-based therapies can have beneficial effects in the treatment of IPF (Table 1). The BLM model is the most used experimental model to study IPF and is considered a well characterized animal model of IPF since it shares many similarities to human IPF pathogenesis [81,82]. However, it must be taken into account that the BLM model does not cover all aspects of the human disease, particularly with regard to the progression of the disease. Therefore, the translatability of preclinical data to clinical efficacy could be limited. In this review, we have focused on a selection of studies conducted in the BLM model since other models of pulmonary fibrosis, such as radiation-induced fibrosis or silica, represent a different disease entity. However, comparisons between all published studies are difficult due to the large number of protocols that have been used. These protocols include different types of stem cells and different routes and times of cell administration after BLM instillation. Among all of these different variables, the timing for cell administration is one of the most critical factors determining the therapeutic outcome. In the BLM model, three developing phases can be distinguished: during the first three days, after the instillation, an inflammatory phase is triggered; after seven days, fibroblasts begin to proliferate; and finally, after 15 days, the fibrosis is already established. Ortiz and colleagues were the first to report that if BM-MSCs were given immediately after BLM instillation, they were engrafted to sites of lung injury, led to less inflammation and collagen deposition and decreased the expression of matrix metalloproteinase (MMP)-2 and MMP-9 (Figure 3) [6]. However, these beneficial effects were not observed if BM-MSCs were given seven days after BLM instillation. Similarly, other studies showed that only the early administration of BM-MSCs (immediately, 15 min, 6, 8, and 24 h, or 3 and 4 days) after BLM instillation were able to show beneficial effects. In these cases, the BM-MSCs were engrafted to the injured tissue, decreased the oedema, decreased the expression of pro-inflammatory and pro-fibrotic cytokines, and decreased the inflammatory cell infiltration, the oxidative stress, the alveolitis, the collagen deposition, the endoplasmic reticulum stress, the ATII cell apoptosis and the fibrosis score (Figure 3) [7,8,9,10,11,12,13,14,15,16]. However, there exist only few studies investigating the administration of BM-MSCs seven or more days after BLM instillation. In these cases, most of the beneficial effects observed when the cells were administered in the early period of BLM instillation are no longer observed (Figure 3) [6,10,17]. There are two studies where BM-MSCs were modified by the transfection of hepatocyte growth factor (HGF) [18] or the overexpression of microRNA (let-7 or miR-154) [19] and were administered after seven days of BLM; these studies showed some positive effects in reducing collagen deposition and fibrosis (Figure 3). All in all, it is clear that the timing of BM-MSCs administration is a determining factor of cell therapy biological response. Related to HSCs, there is only one study that compares HSCs with MSCs as vehicles to deliver keratinocyte growth factor (KGF) into the BLM lungs [11]. Here, Aguilar and colleagues observed that even though both cell populations reduced collagen accumulation, only transduced HSCs transplantation greatly attenuated histological damage. Their conclusion was that the reduced lung damage likely occurs through endogenous ATII cell proliferation induced by KGF [11]. It is important to take into account that in this case, mice underwent a HSCs transplant nine weeks before the BLM instillation, so the cells had already been administered in the animals when fibrosis was induced [11]. However, in this study, it is important to take into account that the authors focused their work on the therapeutic effect of the KGF and not on the delivered cells. In this case, the cells are considered a vector to deliver KGF. However, it is difficult to establish whether the positive effect is due to the cells or to the KGF. In any case, the administration of modified stem cells for the purpose of inhibiting or overexpressing different cytokines or growth factors opens a new field of research.

Adipose-mesenchymal stem cells have also been evaluated in some interesting studies [20,21,22]. Thashiro and colleagues studied the differences between young or old donor adipose-MSCs in old mice after 24 h of BLM instillation [20]. Their results showed that young adipose-MSCs decreased pro-inflammatory markers, fibrosis, MMP-2 activity, oxidative stress and markers of apoptosis (Figure 3). In contrast, old-donor adipose-MSC treatment in old BLM mice did not reduce fibrosis and related markers. This study clearly demonstrates that there are age-dependent anti-fibrotic properties of administered cells [20]. Therefore, the age of the donors is an important issue to take into account in the development of cell therapies for IPF patients, since the results could be different depending on the age of the cell donors. In another study, Kotany and colleagues demonstrated that after seven days of BLM instillation, adipose-MSCs inhibited both pulmonary inflammation and fibrosis, which markedly improved the survival rate of mice in a dose-dependent manner (Figure 3) [21]. In concordance, Lee and colleagues showed by repeated adipose-MSCs administration at 8, 10, 12, and 14 weeks in a chronic model of fibrosis (in which BLM was given every 2 weeks for 16 weeks) a reduced hyperplasia of epithelial cells and reduced inflammation and fibrosis (Figure 3) [22]. These two studies showed that adipose-MSCs could have positive effects even when they are administered in the late periods of BLM instillation.

The studies performed with placenta-MSCs also showed encouraging results when the cells were administered in the early stages of BLM instillation [23,24]. Cargnoni and colleagues investigated the effect of allogeneic or xenogeneic (murine and human) placenta-MSCs after 15 min of BLM instillation [23]. Independently of the cell origin, results were similar. A decrease in neutrophil infiltration and a significant reduction in the severity of fibrosis was observed (Figure 3) [23]. Moreover, in another study, the administration of placenta-MSCs was effective in decreasing pro-fibrotic markers of fibrosis [24]. In related studies, the application of stem cells derived from umbilical or amniotic fluid proved effective, inhibiting the inflammatory response and promoting lung regeneration (Figure 3) [25,26]. Garcia and colleagues showed that amniotic fluid stem cells have the potential to cleave monocyte chemoattractant protein-1 (CCL2), which results in the inhibition of parenchymal remodelling and the development of pulmonary fibrosis even when they have been administered at the same moment or 14 days after BLM (Figure 3) [26].

Few studies have been conducted using iPSCs with or without differentiating them into ATII-like cells [27,28,29]. How and colleagues evaluated two treatments after 24 h of BLM instillation: one with iPSCs and one with a conditioned medium from iPSCs [27]. Notably, both treatments reduced the levels of inflammatory cytokines and chemokines, including interleukin (IL)-1, IL-2, IL-10, tumour necrosis factor α (TNF-α), and collagen deposition (Figure 3). Zhou and colleagues also evaluated iPSCs administration 24 h after BLM instillation [28]. Their results showed a reduction in inflammatory responses, transforming the growth factor β (TGF-β)-1/Smad2/3 pathway and epithelial mesenchymal transition (EMT) during the progression of BLM-induced pulmonary fibrosis (Figure 3) [28].

**Figure 4:**
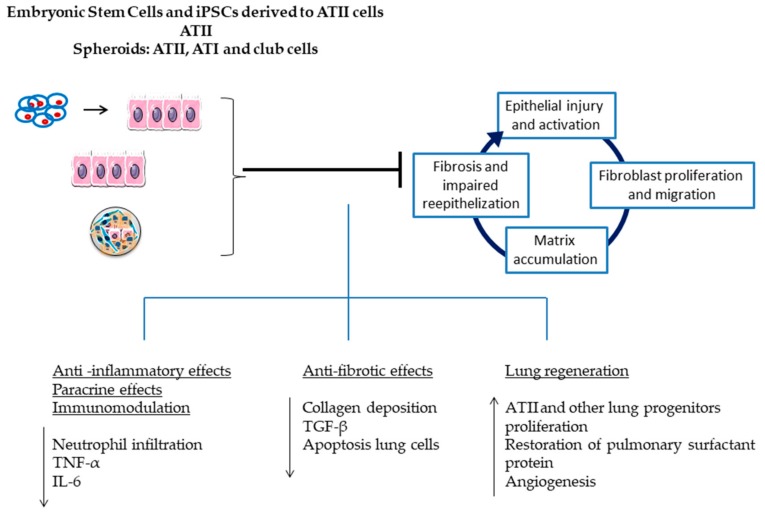
Embryonic stem cells and iPSCs derived to alveolar type II cells, isolated alveolar type II cells and lung spheroids (ATII cells, ATI cells and club cells) delivered intravenously or intratracheally home to the sites of injury in the lungs where they exert anti-inflammatory and anti-fibrotic effects, engage in paracrine signaling and immunomodulation, differentiate into local cell types, and activate resident stem cells enhancing lung regeneration.

Since lung resident stem/progenitor cells are responsible for repairing the damaged alveolar epithelium in the event of lung damage, a number of studies have been performed using lu-MSC, iPSCs and ESCs differentiated into ATII cells, or ATII cells directly [29,30,31,32,33,34]. Zhou and colleagues observed a beneficial effect of iPSCs differentiated into ATII when the cells were administrated 24 h after BLM instillation [29]. Their results showed that the ATII cells differentiated from iPSCs were able to reduce inflammation and fibrosis (Figure 4). Similar results were obtained by Banerjee and colleagues [30]. In this case, they investigated the use of ESCs differentiated into epithelial lineage-specific cells (ATII, ATI, and Club cells). The cell administration was seven days after BLM instillation and a reduction in collagen deposition and an increase in the number of ATII, ATI, and lung progenitor cells was observed (Figure 4) [30]. Another study showed that the administration of lu-MSCs, just after BLM instillation, also attenuated pulmonary fibrosis, mitigated pulmonary arterial hypertension, and decreased inflammatory cell infiltration (Figure 3) [31]. Serrano-Mollar and colleagues conducted two remarkable studies evaluating the therapeutic effect of ATII cells (Figure 4) [32,33]. ATII cells were administrated at three different times (3, 7, and 15 days after BLM instillation). The results of these studies showed that ATII cells halt fibroproliferation at all of these time points. However, the most noteworthy results were the significant reduction of fibrosis and the restoration of surfactant levels 15 days after BLM instillation, during the fibrotic phase [32,33]. A very novel study using lung stem cell spheroids has been performed by Cores and colleagues [34]. This study was based on the development of adult lung spheroid cells as an intrinsic source of therapeutic lung stem cells. The administration of these spheroids just after BLM instillation, decreased the severity of fibrosis, apoptosis, protected alveolar structures, and increased the angiogenesis (Figure 4) [34].

## 4. Clinical Experiences

In recent years, at least three phase 1b non-randomized non-placebo clinical trials involving stem cells and a clinical study using the lung stem/progenitor ATII cells have been published [35,36,37,38]. The main objective of these studies was to evaluate the safety and tolerability of the administered cells. In addition, all of them also evaluated the potential beneficial effect that each one could have. In the first clinical trial, 0.5 × 10^6^ cells/kg of body weight of autologous MSCs isolated from adipose tissue was endotracheally administered into 14 patients diagnosed with mild to moderate IPF; these patients were followed up 12 months after the administration of cells [35]. In a second study, heterologous MSCs isolated from placenta were administered intravenously into eight patients with moderate to severe disease [36]. In this clinical trial, two doses of cells were administered and evaluated (1 × 10^6^ and 2 × 10^6^ cells/kg of body weight) and the patients were monitored for six months [36]. In the third study, heterologous MSCs isolated from BM were administered intravenously into nine patients with mild to moderate disease [38]. In this last study, the authors also administered and evaluated three different cellular doses (20, 100, or 200 × 10^6^ BM-MSCs); the patients were assessed until week 60 and 28 days after the administration of cells [38]. In all of these trials, during the treatment and follow-up period, no serious or clinically significant side effects were observed [35,36,38]. Only the third study reported two non-treatment-related deaths due to IPF progression (disease worsening and/or exacerbation) [38]. Likewise, no studies reported changes in the functional tests of patients or alterations in the quality of life indicators since the beginning of the treatment. The conclusions of these trials were that either endobronchial or intravenous administration of stem cells is safe and well tolerated [35,36,38].

In the other clinical study, ATII cells isolated from lungs of organ donors were administered endotracheally, at a dose of 1000 to 1200 × 10^6^ cells/patient, into 16 patients diagnosed with mild to moderate and progressive disease [37]. In this study, to avoid ATII cell rejection reactions, patients followed immunosuppressive and prophylaxis treatment and the rate of sensitization due to ATII cell administration was similar to that observed in a blood transfusion [37]. No relevant side effects were observed, and 13 of the 16 patients showed stabilization in the functional tests performed throughout the one-year follow-up. Unlike the other three studies, patients walked further in the 6-min walk test and also showed a decrease in the degree of dyspnoea and cough [37]. The conclusions of this study were that ATII cell therapy is safe, well tolerated and with possible beneficial effects.

The limitations of these studies include their small sample size, lack of randomization, and lack of a placebo arm. However, without doubt the encouraging results of these four studies will open the doors to new treatments for IPF.

## 5. Conclusions and Future Perspectives

Preclinical and clinical data support the safety of stem cells and lung stem/progenitor cells as a potential therapy for IPF. The precise mechanisms by which stem cells and lung progenitor cells exert their positive effect are still not clear. These mechanisms involve multiple biological pathways, including engraftment in damaged zones, differentiation into lung epithelial cells, immunomodulatory ability, the secretion of anti-inflammatory and anti-fibrotic mediators, and the promotion of lung endogenous repair. Regarding MSC lung therapies, it is important to note that most studies that have used them have shown that the reparative/healing effects are mostly through the paracrine or immunomodulatory effects on recipient lung tissue, but not by engraftment. Therefore, it would be important to consider MSC therapy as a cell-based immunomodulatory therapy rather than considering it only as a regenerative or reconstituting therapy. On the other hand, the use of allogeneic lung stem/progenitor cells has shown to be a great potential cell therapy option for IPF patients. Moreover, the use of ESCs and iPSCs derived into lung stem/progenitor cells for the repair of the lung after injury and disease condition opens another door towards the development of regenerative therapeutics for lung disease. However, the pluripotent nature of ESCs or iPSCs could present a potential risk of teratogenic effects, which needs to be rigorously addressed before moving into human clinical trials.

Regenerative stem cell therapy has generated a lot of hope amongst scientists and physicians who seek more effective treatment strategies; nevertheless, because different approaches have been applied in preclinical and clinical studies, many key questions remain unanswered. For instance, questions about the effectiveness and efficiency of the route of administration (intravenous or endotracheal) have not yet been resolved. Neither the doses nor the administration timetable nor the stage of the disease at which it is better to start the cell treatment are well defined. Another important question refers to the efficacy on the cell type that should be used, that is, BM-HSCs, BM-MSCs, adipose-MSCs, placenta-MSCs, iPSCs, or lung stem/progenitor cells. Should autologous or heterologous cells be used? Do they really engraft? To answer these important questions, a standardization of protocols should be taken into account. In the majority of preclinical studies, cells have been administered in the early inflammatory phase rather than in the late fibrotic phase. Consequently, the results of most of these studies were positive. By contrast, the effectiveness of stem cell therapies progressively decays as the cells are administered later, when the pro-fibrotic phase or fibrotic phase has already begun. Thus, it could be possible that the positive effects of stem cell therapies are limited only to the inflammatory phase and do not have any effect when fibrosis is already developed. However, with the use of stem cells (that are more differentiated into lung progenitor cells) as ATII cells or even with the use of mature ATII cells, the effectiveness increases even when the cells have been administered during the fibrotic phase. Therefore, both the administration schedule and the cell type should be considered the most important variables for the treatment of IPF.

Although IPF is not recognized as a genetic disease, in recent years, different genome-wide linkage studies have identified genetic polymorphisms that could be associated with higher susceptibility to the development of IPF [83]. Among all mucin 5 (MUC5), telomerase and surfactant protein polymorphisms have been considered the highest genetic risk factor for IPF [83]. Therefore, combining cell-based therapy and gene therapy may offer a new strategy for IPF intervention in the future. In this sense, two different strategies will be important for the development of cell therapy. On the one hand, for autologous cell therapies, it will be important to evaluate the IPF patients’ genome in order to know whether the patients have polymorphisms related to the increased risk of fibrosis. On the other hand, for heterologous cell therapies, it will be important to evaluate the cell donor genome. Finally, another important issue to take into account will be the cell genetic modification, which allows for considerable enhancement of the therapeutic activity. Some preclinical studies have already demonstrated that cells that overexpress or downregulate genes involved in the development of fibrosis could also have positive results restoring damage in pulmonary fibrosis [10,11,15,19].

The use of cell-based therapies to treat IPF is still at the experimental stage and many challenges remain to be explored. Further investigations are necessary to establish the best strategy for using cell-based therapies for the treatment of IPF. Overall, stem cell or lung progenitor cell therapy appears to be a promising strategy to reduce or even reverse pulmonary fibrosis.

## Figures and Tables

**Table 1:** Results of preclinical and clinical studies of cell-based therapies for idiopathic pulmonary fibrosis (IPF).

Study	Cell Source	Dose and Route of Administration	Time of Cell Transplantation after Injury	Study Design	Results	Ref.
Preclinical	BM-MSCs	5 × 10^6^/mouse, IV	Immediately or 7 days after BLM instillation	BLM-induced fibrosis in mice	Reduced inflammation and collagen deposition	[6]
Preclinical	BM-MSCs	2.5 × 10^6^/rat, IV	Immediately or 7 days after BLM instillation	BLM-induced fibrosis in rats	Reduced collagen deposition and reduced oxidative stress	[7]
Preclinical	BM-MSCs	5 × 10^5^/mouse, IV	Immediately after BLM instillation	BLM-induced fibrosis in mice	MSCs protect lung tissue, blocking the pro-inflammatory cytokines TNF-α and IL-1	[8]
Preclinical	BM-MSCs	5 × 10^6^/mouse, IV	6 h after BLM instillation	Myelosuppression and bone-marrow MSC administration. BLM induction in mice	Suppression of inflammation and production of reparative growth factors	[9]
Preclinical	BM-MSCs HGF knockdown BM-MSCs	5 × 10^4^/g body weight, IV	6–8 h after BLM administration or 9 days later	BLM-induced fibrosis in mice	Reduced fibrosis, reduced levels of interleukin-1b and apoptosis, increased levels of HGF; these effects were mediated in part by the HGF	[10]
Preclinical	BM-HSCs MSCs MSCs: transduced (MSCs-KGF) MSCs: non-transduced (MSCs-GFP) HSCs: transduced HSCs with HSCs-KGF HSCs: non-transduced (HSCs-GFP)	0.5 × 10^6^/mouse, IV for MSCs and 0.6 × 10^6^, IV for BM-HSC transplantation	MSCs: 8 h after BLM instillation, second dose 3 days after HSCs: 7 weeks after BM-HSC transplantation BLM instillation + doxycycline	BLM-induced fibrosis in mice and BM-MSC administration. BM-HSC transplantation and BLM-induced fibrosis	MSCs and HSCs reduced collagen disposition. Transduced HSCs attenuated histological damage through endogenous ATII cell proliferation induced by KGF.	[11]
Preclinical	Human BM-MSCs	5 × 10^5^/mouse, IV	24 h after BLM instillation	BLM-induced fibrosis in mice	Reduction of oxidative stress, endoplasmic reticulum stress, and TGF-β1 produced by alveolar cells	[12]
Preclinical	Human BM-MSCs	5 × 10^6^/mouse, IV	1, 2, 3, or 4 days after BLM instillation	BLM-induced fibrosis in immunodeficient NOD/SCID and NOD/SCID/β2 microglobulin (β2M) null mice	Low levels of BM-MSCs engraft	[13]
Preclinical	Hypoxia-preconditioned BM-MSCs	5 × 10^5^/mouse, intratracheal	3 days after BLM instillation	BLM-induced fibrosis in mice	Reduction of inflammation and fibrosis and improved pulmonary function	[14]
Preclinical	Oncostatin M preconditioned BM-MSCs Oncostatin M preconditioned HGF knockdown BM-MSCs	2 × 10^5^/mouse, intratracheal	3 days after BLM instillation	BLM-induced fibrosis in mice	Reduction of inflammation and fibrosis factors and improved pulmonary function	[15]
Preclinical	BM-MSCs	10^6^/rat, IV	4 days after BLM instillation	BLM-induced fibrosis in rats	Reduction of inflammation and fibrosis factors (IL-1β, TGF-β, VEGF, IL-6, TNF-α, and NOS)	[16]
Preclinical	BM-MSCs Amnion-MSCs Human amniotic epithelial cells (hAECs)	1 × 10^6^/mouse, IV	10 days after BLM (72 h after the second BLM dose)	BLM-induced fibrosis in mice (2 repeated doses at 0 days and 7 days)	Amnion-MSCs, BM-MSCs, and hAECs exert a wide range of anti-inflammatory effects. Among all cells, amnion-MSCs were more effective, reducing fibrosis and TGF-β, and increasing MMP-9 activity, GM-CSF secretion and induction of IL-1RA.	[17]
Preclinical	BM-MSCs transfected with HGF	3 × 10^6^/rat, intratracheal	7 days after BLM instillation	BLM-induced fibrosis in rats	Reduced collagen deposition and reduced fibrosis in Ashcroft score	[18]
Preclinical	Human BM-MSCs overexpressing microRNAs let-7d or miR-154	5 × 10^4^/mouse, IV	7 days after BLM instillation	BLM-induced fibrosis in mice	B-MSCs overexpressing let-7d revealed shifts in animal weight loss as well as reduced collagen deposition and decreased CD45-positive cells	[19]
Preclinical	Young-donor adipose-MSCs Old-donor adipose-MSCs	5 × 10^5^/mouse, IV	24 h after BLM instillation	BLM-induced fibrosis in old mice >22 weeks old	Young adipose MSCs showed greater effect on decreased fibrosis, (MMP)-2 activity, oxidative stress, and markers of apoptosis	[20]
Preclinical	Adipose-MSCs	2.5 × 10^4^ or 2.5 × 10^5^/mouse, IV	7 days after BLM instillation	BLM-induced fibrosis in mice	Inhibition of both pulmonary inflammation and fibrosis in a dose-dependent manner	[21]
Preclinical	Human adipose-MSCs	3 × 10^5^/mouse, IP	Were simultaneously administered in the latter 2 months of the 4-month BLM regimen at the same time of BLM	Biweekly administration of a total of 8 doses of BLM during 4 months in mice	Reduced epithelial cell hyperplasia and reduced inflammatory cell infiltration and fibrosis. Inhibition of apoptosis in epithelial cells and in the expression of TGF-β)	[22]
Preclinical	Human placenta-MSCs Murine placenta-MSCs	4 × 10^6^/mouse, IP 1 × 10^6^/mouse, IV or intratracheal	15 min after intratracheal BLM instillation	BLM-induced fibrosis in mice	Reduction in neutrophil infiltration and in the severity of BLM-induced lung fibrosis	[23]
Preclinical	Human placenta-MSCs	1 × 10^5^/mouse, IV	3 days after BLM instillation	BLM-induced fibrosis in MyD88-deficient mice	Reduced collagen deposition, MyD88 and TGF-β signalling activation, and production of pro-fibrotic cytokines	[24]
Preclinical	Human umbilical-MSCs	1 × 10^6^/mouse, IV	24 h after BLM instillation	BLM-induced fibrosis in mice	Inhibition of inflammation and fibrosis and down-regulation of lung cytokine and TIMP expression while up-regulating MMPs	[25]
Preclinical	Amnion stem cells (ASCs)	5 × 10^6^/mouse, IV	2 h after BLM instillation, 0 or 14 days after BLM instillation	BLM-induced fibrosis in mice	Inhibition of collagen deposition, preservation of pulmonary function, and decreased CCL2 expression on either day 0 or day 14	[26]
Preclinical	iPSCs iPSC conditioned medium	2 × 10^6^/mouse, IV	24 h after BLM instillation.	BLM-induced fibrosis in mice	Decreased myeloperoxidase activity and neutrophil infiltration. Rescue of pulmonary function. Reduced collagen deposition	[27]
Preclinical	iPSCs	2 × 10^5^/mouse, IV	24 h after BLM instillation	BLM-induced fibrosis in mice	Suppression of inflammatory responses, the TGF-b1/Smad2/3 pathway, and EMT	[28]
Preclinical	iPSCs derived to ATII cells	5 × 10^5^/mouse, intratracheal	24 h after BLM instillation.	BLM-induced fibrosis in mice	Reduced lung inflammation and collagen deposition	[29]
Preclinical	Human- ESCs derived to epithelial lineage-specific cells (ATII, ATI and club cells)	10^5^ Human-ESC, intratracheal	7 days after BLM instillation	BLM-induced fibrosis in mice	Reduced collagen and increased levels of ATI and ATII and progenitors in the lungs	[30]
Preclinical	Lung resident-MSCs	0.15 × 10^6^ or 0.25 × 10^6^/mouse, IV	Immediately after BLM instillation	BLM-induced fibrosis in mice	Decreased pulmonary damage and mitigation of the development of PAH. Decreased lymphocyte and granulocyte infiltration	[31]
Preclinical	ATII cells	2.5 × 10^6^/rat, intratracheal	3, 7 or 14 days after BLM	BLM-induced fibrosis in rats	Reduced collagen deposition and reduced severity of pulmonary fibrosis	[32]
Preclinical	ATII cells	2.5 × 10^6^/rat, 14 days after BLM	14 days after BLM	BLM-induced fibrosis in rats	Restoration of lung surfactant protein levels	[33]
Preclinical	LSCs formed basically by ATI, ATI and club cells	5 × 10^6^ LSC/rat, IV	At the same moment of intratracheal BLM instillation	BLM-induced fibrosis in rats	LSCs attenuated the progression and severity of fibrosis, decreased apoptosis, protected alveolar structures, and increased angiogenesis	[34]
Clinical	Autologous adipose-MSCs	0.5 × 10^6^ cells/kg of body weight, intratracheal	Mild to moderate IPF patients	Phase1b, prospective, non-randomized, non-placebo (*n* = 14)	Adipose-MSCs were safe and no deterioration of functional parameters and indicators of quality of life were observed	[35]
Clinical	Heterologous placenta-MSCs	1 × 10^6^ or 2 × 10 ^6^ cells/kg of body weight, IV	Mild to moderate IPF patients	Phase 1b, non-randomized, non-placebo, dose escalation study (*n* = 8)	Placenta-MSCs were safe, with no evidence of worsening fibrosis	[36]
Clinical	Heterologous ATII cells	1000 to 1200 × 10^6^ cells/patient, intratracheal	Mild to moderate IPF patients	Clinical study, non-randomized, non-placebo (*n* = 16)	ATII cells were safe and well tolerated, and halted disease progression	[37]
Clinical	Heterologous BM-MSCs	20, 100, or 200 × 106 cells/patient, IV	Mild to moderate IPF patients	phase 1b, non-randomized, non-placebo, dose escalation study (*n* = 9)	BM-MSCs were safe, no evidence of worsening fibrosis	[38]

Abbreviations: BM, bone marrow; MSCs, mesenchymal stem cells; HGF, hepatocyte growth factor; HSCs, hematopoietic-stem cells; GFP, green fluorescent protein; KGF, keratinocyte growth factor; NOD/SCID, nonobese diabetic/severe combined immunodeficiency; AEC, amniotic epithelial cells; RA, receptor antagonist; MYD88, myeloid differentiation primary response 88; iPSCs, induced pluripotent stem cells; ESCs, embryonic stem cells; ATII cells, alveolar type II cells; ATI, alveolar type I cells; LSCs, lung spheroid cells; IV, intravenously; IP, intraperitoneally; BLM, bleomycin; TNF-α, tumour necrosis factor-α; IL, interleukin; TGF-β, transforming growth factor-β; VEGF, vascular endothelial growth factor; NOS, nitric oxide; MMP, metalloproteinases; GM-CSF, granulocyte macrophage colony-stimulating factor; TIMP, tissue inhibitor of metalloproteinases; CCL2, monocyte chemoattractant protein-1; EMT, epithelial to mesenchymal transition; PAH, pulmonary arterial hypertension.
